# P-65 - UNDERSTANDING THE BURDEN OF MYCOBACTERIUM TUBERCULOSIS IMMUNOREACTIVITY IN URBAN AND RURAL POPULATIONS: A SYSTEMATIC REVIEW, META-ANALYSIS, AND EPIDEMIOLOGICAL ANALYSIS

**DOI:** 10.1093/pubmed/fdag046.090

**Published:** 2026-07-09

**Authors:** Miss Emma Mckinlay, Dr Peter MacPherson

**Affiliations:** University Of Glasgow; University Of Glasgow

## Abstract

**Background:**

Tuberculosis (TB) is the leading cause of death from a single infectious pathogen, responsible for one million deaths annually, principally in low-and-middle income countries (LMICS). TB exists as an active disease, which is symptomatic and transmissible and as a latent infection which is asymptomatic but includes risk of activation. Transmission and access to healthcare vary between urban and rural settings: overcrowding may drive urban transmission, while barriers to diagnosis and treatment exist in rural populations.

**Objectives:**

Investigate the burden of latent TB in urban and rural populations in LMICs using meta-analysis to pool estimates of TB prevalence and understand temporal trends. Evidence based policy recommendations will then be made.

**Methods:**

Data was obtained from a prior PhD on TB. Studies reporting urban-and-rural prevalence data were included and appraised using the Hoy et al risk of bias tool. Descriptive statistics were used to illustrate the crude urban-to-rural TB ratio. A random-effects meta-analysis was performed to generate pooled estimates. Meta-regression was carried out to analyse temporal trends. All analysis was done in R.

**Results:**

Eleven studies (five national, six subnational) met the inclusion criteria. Crude urban-to-rural ratios ranged from 0.78 to 56.5, indicating considerable heterogeneity. Most studies showed similar or moderate differences between the urban and rural prevalence. However, surveys from Sudan and Sri Lanka were outliers with a substantially higher urban prevalence. The meta-analysis showed no statistically significant overall difference (OR 1.69, 95%CI 0.83-3.42 p<0.001).

**Conclusions:**

No consistent global pattern of urban-rural TB prevalence was found. The variability identified reflects the impact of area-specific factors, such as disparities in access to healthcare in rural regions and increasing urban population density.

These findings illustrate the importance of tailored public health strategies to control TB rather than a uniform approach in every region and a need for more epidemiological data to guide public health policy.

